# PANTAX: a phase Ib clinical trial of the efflux pump inhibitor SCO-101 in combination with gemcitabine and *nab*-paclitaxel in non-resectable or metastatic pancreatic cancer

**DOI:** 10.1007/s10637-025-01526-7

**Published:** 2025-04-24

**Authors:** Susy Shim, Anke Reinacher-Schick, Anna-Lena Kraeft, Per Pfeiffer, Line Schmidt Tarpgaard, Thomas Jens Ettrich, Angelika Kestler, Signe Christensen, Haatisha Jandu, Mubeen Nawabi, Nicklas Lindland Roest, Lars Damstrup, Peter Michael Vestlev, Nils Brünner, Jan Stenvang, Morten Ladekarl

**Affiliations:** 1 Department of Oncology, Clinical Cancer Research Center, Aalborg University Hospital, Hobrovej 18 - 22, 9000 Aalborg, Denmark; 2 Department of Clinical Medicine, Aalborg University, Aalborg, Denmark; 3 Dept. of Hematology, Oncology and Palliative Care, St. Josef-Hospital, Bochum, Germany; 4 Department of Oncology, Odense University Hospital, Odense, Denmark; 5 University of Southern Denmark, Odense, Denmark; 6 Department of Internal Medicine I, University Hospital of Ulm - Oberer Eselsberg, Ulm, Germany; 7 Scandion Oncology A/S, Copenhagen, Denmark

**Keywords:** Pancreas cancer, Gemcitabine, Nab-paclitaxel, Chemotherapy resistance mechanisms, Cytotoxicity assay

## Abstract

De novo or acquired resistance to chemotherapy is ubiquitous in pancreatic ductal adenocarcinoma (PDAC). SCO-101 is an oral compound that may counteract chemo-resistance by interacting with SRPK1, ABCG2 drug transporter, and liver enzyme UGT1A1. We first conducted preclinical experiments in paclitaxel-resistant PDAC cells to access the tumoricidal effects of SCO-101 or SRPK1-inhibitor alone or in combination with paclitaxel. Second, we enrolled 22 patients with non-resectable PDAC in a phase Ib trial to investigate safety and pharmaco-kinetics, and to establish maximum tolerated dose (MTD) by evaluation of dose-limiting toxicities (DLTs) during the first cycle of 80% dose gemcitabine (Gem) and *nab*-paclitaxel (Nab) together with increasing doses of SCO-101. In paclitaxel-resistant PDAC cells in vitro, a synergistic effect between SCO-101 and paclitaxel was demonstrated. In patients, daily doses for 6 days of SCO-101 resulted in a two- to threefold drug accumulation, and drug exposure was dose proportional. Treatment was well tolerated. Transiently increased blood bilirubin attributable to SCO-101 was observed in 12 cases (55%) and associated with jaundice in three patients. One and two DLTs, respectively, were observed at 150 and 250mg dosing-levels of SCO-101, and the MTD was determined to be 200 mg of SCO-101 daily for 6 days on a bi-weekly schedule together with 80% dose of Gem and Nab. Median progression-free and overall survival was 3.3 and 9.5 months, respectively. In PDAC, SCO-101 can be added to Gem and Nab with little and manageable toxicity. However, no clear added efficacy signal was observed of the combination. Trial registration number: NCT04652205 (Nov 29, 2020).

**Supplementary Information:**

The online version contains supplementary material available at 10.1007/s10637-025-01526-7.

## Background

Pancreatic ductal adenocarcinoma (PDAC) is one of the most lethal and aggressive malignancies. Half a million cases were diagnosed worldwide in 2020 [2], and PDAC is projected to become the third leading cause of cancer death in Europe by 2025 [3]. Curatively intended surgery can only be offered to 15–20% of patients but these patients are at high risk of relapse [4]. Therefore, the treatment goal for the vast majority is improvement of quality of life and prolonged survival.

Among the most used systemic treatments for inoperable PDAC is the combination of gemcitabine (Gem) and *nab*-paclitaxel (Nab) [4]. The treatment regimen was introduced in 2013 as a result of the MPACT phase III study including 861 patients with metastatic PDAC [5]. Patients were randomized 1:1 to single-drug Gem or the combination of Gem and Nab. The objective response rate (ORR) with Gem and Nab was 23% as opposed to 7% with Gem alone, and the median overall survival (mOS) improved significantly from 6.7 to 8.5 months (hazard ratio (HR), 0.72; 95% confidence interval (CI) 0.62–0.83; *P* < 0.001). However, as with any type of chemotherapy used in PDAC, a high frequency of treatment resistance was observed. Twenty percent showed progression at the first evaluation at week 8, while half of the patients had progressed after 5.5 months and the majority (84%) after 1 year [5]. Based on these dismal figures, mechanisms of drug resistance and drugs that may revert or delay resistance have been the focus of numerous research projects [6].

SCO-101 is a small molecule for oral use under clinical development as an inhibitor of drug resistance [7]. SCO-101 has been shown to counteract resistance to irinotecan by inhibiting the drug efflux pump ATP-binding cassette (ABC) G2 and the metabolic liver enzyme UDP glucuronosyltransferase (UGT)1A1 [8]. While neither Gem nor Nab is considered to be a substrate for ABCG2 [9] or UGT1A1 [10], preclinical evidence suggests that ABCG2 may play an important role in PDAC progression and resistance to Gem by indirect mechanisms [11–13], and also the serine-arginine protein kinase 1 (SRPK1), which is a potential therapeutic target in PDAC [14, 15], was recently shown to be targeted by SCO-101 [16]. In further support of a role of SCO-101 together with taxane-based combination chemotherapy, we previously showed that SCO-101 could re-sensitize taxane-resistant breast cancer cells to taxane in an in vitro model [17].

Phase I studies with SCO-101 monotherapy in healthy volunteers showed low toxicity [18]. Subjects exposed to single doses up to 200 mg or multiple doses up to 150 mg daily for 14 days showed mild adverse events (AEs) and no serious adverse events (SAEs). The most frequently reported treatment-emergent AE was transient headache, while blood levels of unconjugated bilirubin showed a transient dose-dependent increase due to a SCO-101-induced, reversible inhibition of the UGT1A1 enzyme. Two individuals in the 150 mg dose cohort developed clinical jaundice [18].

In the present study, we first developed a human paclitaxel-resistant PDAC cell line and investigated the growth inhibitory effect of combining either SCO-101 and paclitaxel or SRPK1-inhibitors and paclitaxel. Based on the preclinical results and prior phase I studies, we performed a clinical phase Ib trial in PDAC patients where SCO-101 was combined with Gem and Nab. The primary aims were to investigate safety and toxicity with increasing doses of SCO- 101 and to establish the maximum tolerated dose (MTD) during the first treatment cycle of Gem and Nab together with SCO-101. Moreover, relevant pharmacokinetic parameters were established.

## Methods, materials, and patients

### Preclinical study

A paclitaxel-sensitive human PDAC cell line, PANC-1, was obtained (ATCC, CRL-1469) and the derived paclitaxel-resistant PANC-1-Pac cell line was established by gradually increasing concentrations of paclitaxel over a period of 2–4 months. Standard MTT and crystal violet assays were applied to evaluate the effect of treatment with either combinations of SCO-101 and paclitaxel or SRPK1-inhibitor and paclitaxel. Details are presented in the Online Resource.

### Clinical study design

The PANTAX trial was a prospective, international multicenter phase Ib open-label, non-randomized, dose-escalation study aiming to determine the MTD, safety, and tolerability of SCO-101 when combined with Gem and Nab in patients with non-resectable or metastatic PDAC (Protocol nr. SCO101-002, EudraCT nr. 2020–002627-11; ClinicalTrials.gov identifier NCT04652206). Furthermore, the study aimed to establish the pharmacokinetic profile of SCO-101 in combination with Gem and Nab. ORR, clinical benefit rate (CBR), median progression free survival (mPFS), and mOS were included as secondary end points.

Patients were recruited at four centers, two in Denmark and two in Germany. It was a standard 3 + 3 dose escalation study with increasing doses of SCO-101 and a fixed (80%) start dose of Gem and Nab. At least three consecutive patients were sequentially included at each SCO-101 dose level. Dose-limiting toxicities (DLTs), defined as shown in Table 1, were evaluated during the first chemotherapy cycle of 28 days. If one patient of a cohort had a DLT, a further cohort of three patients was treated with the same dose level without escalating the dose. If only one out of the six patients at this dose had a DLT, the trial continued as planned at the next higher dose level. If two or more patients in a cohort had a DLT, the dose escalation stopped at that level and the prior lower dose cohort was expanded to include a total of six patients. If a maximum of one patient in the expanded cohort exhibited a DLT, this dose level was considered the MTD.

**Table 1:** Adverse events used for definition of dose-limiting toxicity during the first cycle of treatment*

• Death
• Anaphylactic reaction to SCO-101 when given in the first five days of a treatment period before treatment with Gem and Nab
• Any life-threatening SAE which is not described in the current version of the Summary of Product Characteristics for Gem and Nab [19]
• Grade 4 laboratory abnormalities, including but not limited to grade 4 thrombocytopenia (platelet count < 25 × 10^9^/L), grade 4 neutropenia > 7 days (absolute neutrophil count < 0.5 × 10^9^/L) and grade 4 blood bilirubin increase (> 10 × ULN), or a clinically significant increase (> 20 μmol/L) in conjugated bilirubin not related to obstruction of the bile duct system or cholangitis
• Febrile neutropenia
• Grade ≥ 3 non-hematologic toxicity, except grade 3 fatigue of less than one week duration, and grade 3 nausea, vomiting and diarrhea that resolve within 48 h following institution of appropriate supportive care with anti-nausea and anti-diarrheal agents
• AEs judged to be possibly, probably or definitely related to receipt of SCO-101 in combination with Gem and Nab and leading to delay of treatment for more than 2 weeks

^*^If a causal (possible, probable or definite) relation to SCO-101 is assessed by the investigator at the treatment site

*AE* adverse event, *Gem* gemcitabine, *Nab nab*-paclitaxel, *SAE* serious adverse event, *ULN* upper limit of normal

### Patients

Inclusion and exclusion criteria are shown in Online Resource (Supplementary Table 1). In short, patients included were in Eastern Cooperative Oncology Group (ECOG) Performance Status (PS) 0–2 with histologically or cytologically verified non-resectable or metastatic PDAC, not amenable for curatively intended treatment but eligible for treatment with standard dose Gem and Nab.

### Study treatment

**Fig. 1:**
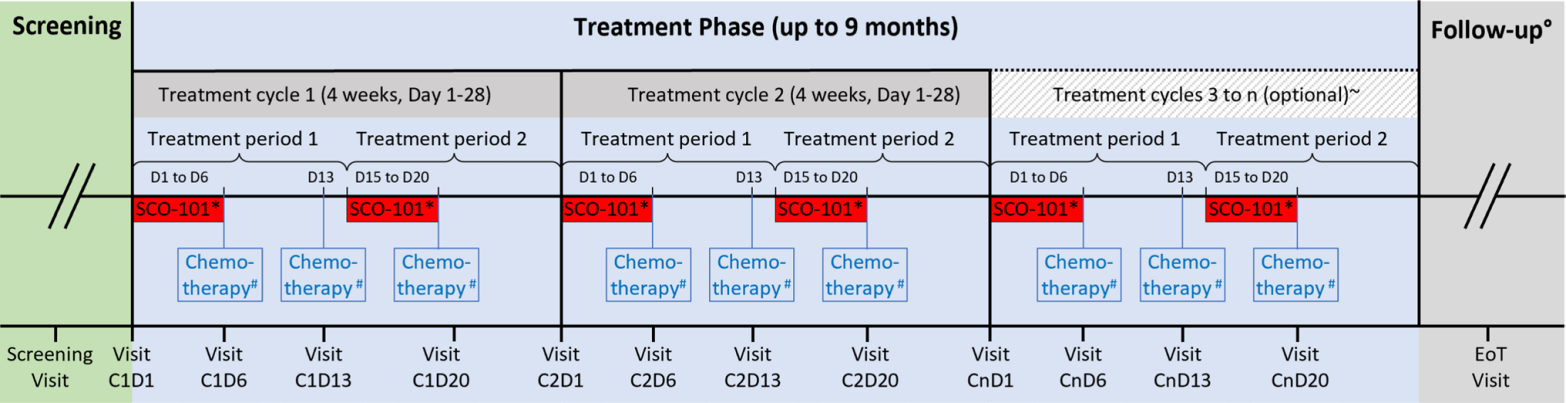
Study treatment including screening, treatment phase, and follow-up. Visits are indicated on x-axis. *SCO-101 orally for 6 days before chemotherapy at day (D) 6 and 20; # gemcitabine and *nab*-paclitaxel with a starting dose of 80% recommended dose; ~ SCO-101 treatment for a maximum of 9 months; ^o^ follow-up until end of study or death. EoT, end of treatment

Study treatment is illustrated in Fig. 1. SCO-101 was dosed once daily on days 1–6 and 15–21 before chemotherapy every 4 weeks (28 days, one cycle) with a starting dose of 50 mg. Chemotherapy with Gem and Nab was given days 6, 13, and 20 in each cycle. The SCO-101 dose in succeeding cohorts was escalated with 50 mg increments to a maximum of 350 mg if no DLTs were observed within the first cycle of chemotherapy in prior cohorts. Based on available PK-data [18], the treatment schedule of SCO-101 was designed to obtain a systemic concentration of SCO-101 that would be biologically active during at least two out of three Gem and Nab treatments but would allow for SCO-101 effects to subside before the next chemotherapy was initiated. During the first cycle, patients received treatment with chemotherapy at a dose corresponding to 80% of the recommended dose [19], i.e., 100 mg/m^2^ Nab and 800 mg/m^2^ Gem given as infusions over 30 min. If the first treatment cycle was well tolerated (no toxicity above grade 1), the investigator had the option in the following treatment cycles to increase the dose of chemotherapy up to 100%. The dose of chemotherapy was further adjusted to the patient´s tolerability in accordance with the chemotherapy-product monographs [19]. Standard antiemetics were allowed as was the use of granulocyte colony-stimulating factor (G-CSF) in case of treatment-induced neutropenia. If chemotherapy was delayed because of toxicity, SCO-101 was paused. At re-exposure, SCO-101 was dosed daily for 6 days before continued chemotherapy. Treatment continued until progressive disease (PD), intolerance, or study withdrawal; however, SCO-101 could be administered for a maximum of 9 months.

### Ethical considerations

The study was conducted in accordance with the Declaration of Helsinki, Good Clinical Practice Guidelines, and relevant Institutional Review Board and Ethics Committee requirements (Ethics committee number N20200048 (Denmark) and 154/21 (Germany)). All patients provided written informed consent to participate.

### Assessment

Toxicity was assessed at baseline and weekly at days 1, 6, 13, and 20 before each treatment. Toxicities were recorded and graded according to the Common Terminology Criteria for Adverse Events (CTCAE) version 5.0 [20], and safety and tolerability of SCO-101 in combination with Gem and Nab were determined by number, frequency, and severity of AEs collected from the time of first treatment with SCO-101 until end of treatment. All SAEs were registered from consent and were followed for 30 days after end of treatment with SCO-101. The relationship of AEs with SCO-101 was assessed by local investigators as unrelated, unlikely related, possible, probable, or definitely related.

### Pharmacokinetics

Pharmacokinetics (PK) blood samples were drawn during the first cycle of treatment on day one of SCO-101 administration (6 time points), on day six (6 time points), and on day nine (72 h after last SCO-101 treatment). Plasma samples were analyzed for SCO-101 using a bioanalytical method (BM/2020/0998) developed and validated under study KF64YR at LabCorp (England). SCO-101 was extracted from human plasma by protein precipitation and analyzed by liquid chromatography using tandem mass spectrometry (LC–MS/MS). Pharmacokinetic parameters were determined by noncompartmental analysis using the software package SAS (version 9.4 or higher). The PK profile of SCO-101 was evaluated by maximum drug plasma concentration (C_max_) and area under the plasma drug concentration–time curve (AUC_0 to 8 h_).

### Efficacy

Tumor evaluation by CT and MRI scans were done every 8 weeks. Response was determined according to RECIST v 1.1 [21] by local assessment. ORR was determined as the percentage of evaluable patients experiencing a complete response (CR) or partial response (PR). CBR was determined as the percentage of evaluable patients experiencing response or stable disease (SD) for over 16 weeks. Date of study inclusion was the index date. For assessment of PFS, the date of PD was the date of imaging showing progression or date of last treatment in patients with deteriorating clinical condition. Patients not followed until progression were censored for PFS at the date of last follow-up. One patient was lost to follow-up for survival and was censored from OS analysis on the date of last visit. End of study was 6 months after the last patients last treatment visit.

### Statistical analysis

Statistical analyses were mainly descriptive and included mean, median, range, and 95% CIs. Kaplan–Meier plots were constructed using Stata version 18 (StataCorp, 2023). In the preclinical study, the zero interaction potency (ZIP) synergy score was calculated as described by Yadav et al. [22], to quantify the deviation from the expectation of zero interaction between drugs. A ZIP synergy score above 10 indicates synergy [23].

## Results

### Preclinical study

**Fig. 2:**
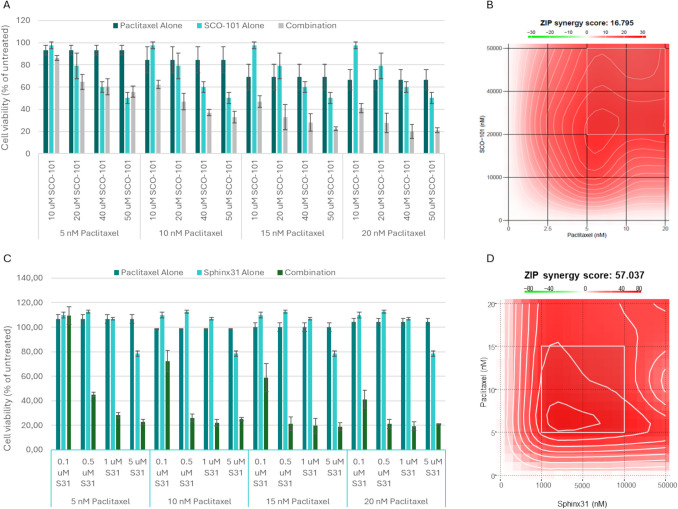
Synergistic effect in Panc-1-Pac cells when combining paclitaxel with SCO-101 or SRPK1-inhibitor. **A** Cell viability. Effect of treatment with paclitaxel and SCO-101 alone and in combination for 72 h with drug + 72 h with fresh media without drugs. An experiment with individual treatments in triplicate is shown. Values are represented as percentage normalized to untreated cells. Error bars indicate standard deviation. **B** Visualization of synergy scores for SCO-101/paclitaxel combinations. The mean ZIP synergy score was 16.795, indicating synergy between SCO-101 and paclitaxel. **C** Cell viability. Effect of treatment with paclitaxel and SPINX31 (S31) alone and in combination for 144 h. An experiment with individual treatments in triplicate is shown. Values are represented as percentage normalized to untreated cells. Error bars indicate standard deviation. **D** Visualization of synergy scores for SPHINX31/paclitaxel. The mean ZIP synergy score was 57.037, indicating synergy between SPHINX31 and paclitaxel

A paclitaxel-resistant human PANC-1 PDAC cell line was established, named PANC-1-Pac, and the resistant phenotype relative to the parental PANC-1 control cell line was five times higher in the resistant cell line (Online Resource: Supplementary Fig. 1). As shown in Fig. 2, both SCO-101 and paclitaxel had a dose-dependent effect on the PANC-1-Pac cells and a stronger effect was observed when SCO-101 was combined with paclitaxel, especially at the higher concentrations. Synergy between SCO-101 and paclitaxel was found (ZIP synergy score 16.795). The combination effect in the PANC-1-Pac cells was verified by another assay, whereas in the wild-type PANC-1 cells, no combination effect of SCO-101 and paclitaxel was observed (Online Resource: Supplementary Fig. 2). SCO-101-inhibition of SRPK1 was analyzed by two different kinase activity assays and the half-maximal inhibitory concentration (IC50) values were either 2.4 or 3.0 µM [8]. The combination of paclitaxel and the SRPK1-inhibitor SPHINX31 caused synergistic effects on cell viability (ZIP synergy score 57.037). The synergistic effect of paclitaxel and SRPK1-inhibition was verified by another SRPK1-inhibitor, SRPIN340 (ZIP synergy score 21.4) (Online Resource: Supplementary Fig. 3).

### Clinical study

#### Patients and treatment

Thirty-two patients were screened, and 22 evaluable patients were enrolled between June 2021 and February 2023. Median age of patients was 65 years, ranging from 51 to 77 years. Additional baseline characteristics are shown in Online Resource, Supplementary Table 2. In short, all patients were in ECOG PS 0–1 except one. Nineteen (86%) patients had metastatic disease, and 17 patients (77%) had previously completed other types of chemotherapy prior to Gem and Nab. Of note, 16 patients had received FOLFIRINOX. Being allowed per protocol, five patients had received up to one cycle of Gem and Nab before entering the study.

Concomitant SCO-101 and chemotherapy was administered for a median of 9 weeks (range 1 to 37 weeks), while chemotherapy alone was administered for a median of 3 additional weeks. Except for three patients who went off study after experiencing a DLT in their first treatment cycle, all patients proceeded treatment with SCO-101 and chemotherapy beyond the first treatment cycle. No patients had chemotherapy dose escalated to 100%.

#### Safety and tolerability

The maximum grade of AEs, assessed by investigators to be possibly, probably, or definitely related to SCO-101, is shown in Table 2. All patients experienced at least one such AE. Twenty (91%) patients experienced an AE ≥ grade 3, and two (9%) patients experienced an AE grade 4 (thrombocytopenia in both cases). The vast majority of recorded adverse effects were as expected with Gem and Nab alone [19]. However, elevation of blood bilirubin—a previously described effect of SCO-101 [18]—was observed in 12 (55%) cases and associated with clinical jaundice in three cases. The elevation in bilirubin was not accompanied by biochemical hepatotoxicity and resolved within 1 week (data not shown). In addition, one case each of increased blood lactate AE grade 3 at dose level 50 mg SCO-101 and of thrombotic thrombocytopenic purpura (TTP) AE grade 3 at dose level 150 mg SCO-101 was registered. TTP, although rare, has been associated with administration of chemotherapy including Gem in prior reports [24].

**Table 2:** Treatment-related adverse events occurring during treatment with SCO-101 plus gemcitabine and *nab*-paclitaxel*

	Cohort 1 50 mg SCO-101		Cohort 2 100 mg SCO-101		Cohort 3 150 mg SCO-101		Cohort 4 200 mg SCO-101		Cohort 5 250 mg SCO-101		Sum of cohorts	
No patients	3		3		6		6		4		22	
CTC-AE grade^a^	Any	≥ 3	Any	≥ 3	Any	≥ 3	Any	≥ 3	Any	≥ 3	Any	≥ 3
Type of AE												
Nausea	3		3		2		3		2		13	
↑B-bilirubin	2		1		2	1	4		2		12	
Fatigue	3		1		2	1	4		2		12	1
↓B-hemoglobin	2		1		4	2	3		1		11	2
Diarrhea	3		3		3		2				11	
Alopecia	2		2		2		4				10	
↓B-thrombocytes	3				1		2		2	2	8	2
Rash^b^	2		2		1		2		1		8	
↓B-neutrophils	3	3	1		1		1	1	1	1	7	5
Stomatitis			1		2		4				7	
Paraestesia	1		2	1			1		3		7	1
Pain	2				1		2				5	
↓B-lymphocytes	3	2									3	2
Candidiasis			1		1				1		3	
Constipation					1		1		1		3	
Vomitting					2		1				3	
Dysgeusia			1				1		1		3	
Jaundice					1	1	1		1		3	1
Headache	1				1		1				3	
Oedema			1				1				2	
Athralgia	1				1						2	
Nail disorder	1		1								2	
Loss of appetite							2				2	
Dizziness	1						1				2	
TTP					1	1					1	1
↑B-lactate	1	1									1	1
Hypertension	1	1									1	1
Chills	1	1									1	1

^*^If a causal (possible, probable or definite) relation to SCO-101 was assessed by the investigator at the treatment site. AEs < grade 3 occurring in only one case are not shown

*AE* adverse event, *B* blood, *CTC* Common Terminology Criteria, *TTP* thrombocytopenic thrombotic purpura

^a^Maximum grade registered per patient

^b^Includes three cases of maculo-papular rash and acneiform dermatitis each, and one case of folliculitis, urticaria, and eczema each

No suspected unexpected serious adverse reactions (SUSARs) were recorded. Nineteen SAEs were noted among 11 patients; however, only one was deemed related to SCO-101 (transiently increased blood bilirubin AE grade 3 at 150 mg SCO-101 dose level). One patient at the 200 mg dose level experienced acute pancreatitis after cycle 6 and succumbed 3 weeks later from the condition, which was deemed unrelated to treatment.

#### DLT and MTD

One DLT (blood bilirubin of 24 µmol/L) was observed at dose level 3 (150 mg SCO-101) and this cohort was therefore expanded to six patients, but with no further DLTs observed. No DLTs were observed among three patients at dose level 4 (200 mg SCO-101), while two DLTs (two cases of thrombocytopenia AE grade 4) were observed at dose level 5 (250 mg SCO-101). As no further DLTs were observed after expansion of the level 4 cohort, the 200 mg dosing level of SCO-101 was the MTD.

#### Pharmacokinetics

**Fig. 3:**
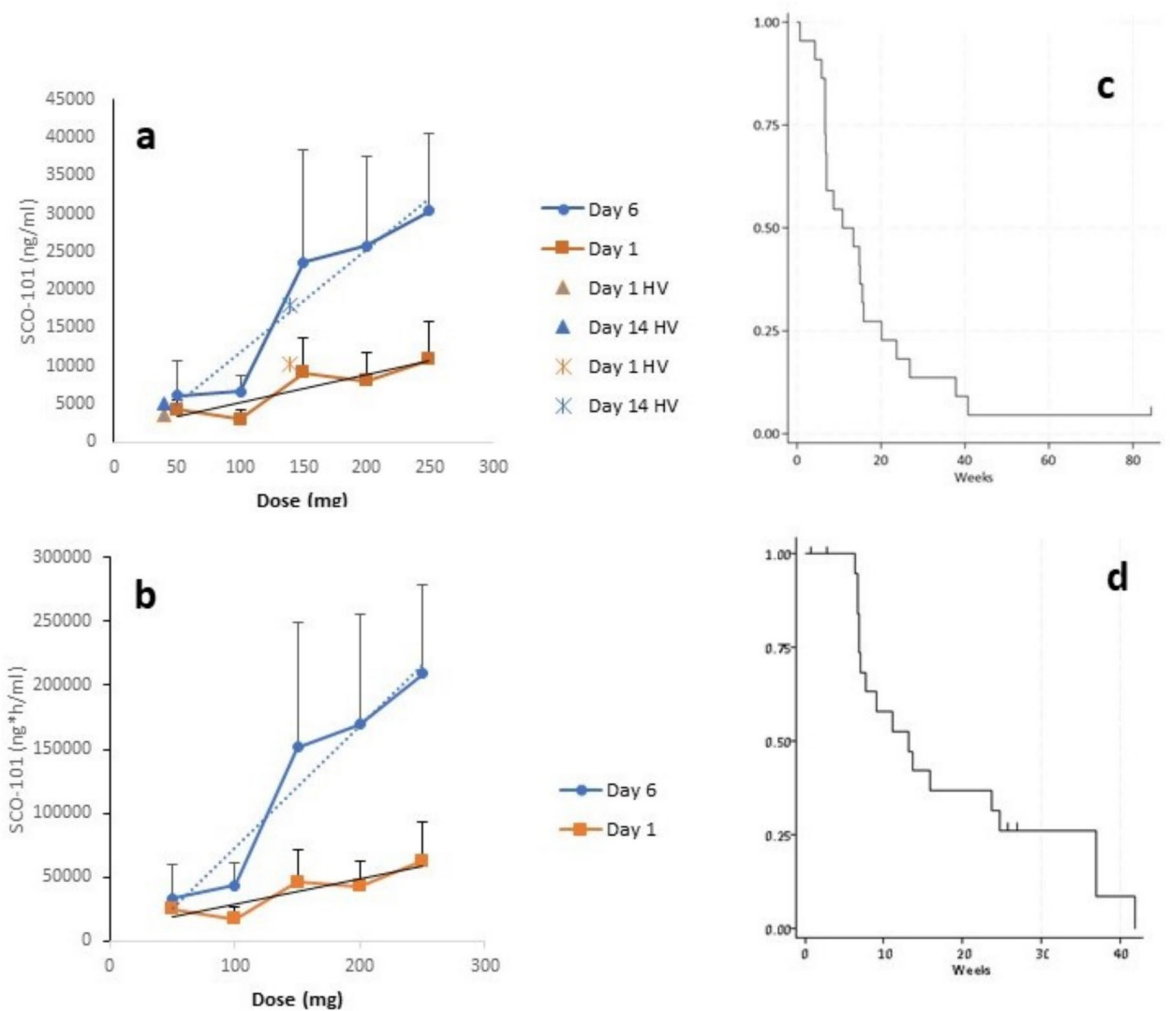
Pharmacokinetics and clinical outcome of SCO-101 and 80% dose of Gem and Nab. **a** Maximum drug plasma concentration, C_max_, and **b** area under the plasma drug concentration–time curve, AUC_0-8 h_, of six daily doses of SCO-101. Daily dose of SCO-101 is shown on x-axis and plasma SCO-101 measurements on the y-axis. For comparison, C_max_ exposures previously measured in a phase I study with healthy volunteers (HV) following repeated dosing of 50 mg and 150 mg SCO-101 for 14 days [18] have in **a** been extrapolated to 40 mg and 140 mg to better illustrate the similar and overlapping exposures. The AUC_0 to 8 h_ was not calculated in the HV phase I study, and therefore it is not shown in **b**. **c** Kaplan-Meyer plot of progression-free survival, and **d** overall survival of patients. Patients censored are indicated by vertical bars

Daily doses for 6 consecutive days of SCO-101 resulted in a two- to threefold accumulation for both C_max_ and AUC_0-8 h_ (Fig. 3a and b). The increases in exposures were approximately dose proportional. The blood sampling schedule did not allow for half-life (T_½_) determinations; however, on average, 33% of SCO-101 C_max_ was present at day 9 for the 200 mg and 250 mg doses of SCO-101.

#### Efficacy

Three patients had unmeasurable disease, three patients went off study, and one patient died before the first evaluation, leaving 15 patients evaluable for response assessment. One patient had a PR and six had SD (two of these with near PR) as best response, accounting for an ORR of 6.7%. As one PR was observed, and four patients had SD lasting more than 16 weeks, the CBR was 33%. Eight of 15 patients (53%) with elevated cancer antigen (Ca) 19–9 at treatment initiation had a reduction of serum Ca 19–9 of at least 25% during study treatment, and in two patients Ca 19–9 normalized. The mPFS was 3.3 months (CI 1.7–9.2 months) and mOS was 9.5 months (CI 5.7–16 months) (Fig. 3c and d). At the time of data lock, four patients were alive and all, but one patient had progressed.

## Discussion

The primary objective of this clinical phase Ib study was to investigate safety and tolerability and to establish the MTD for SCO-101 in combination with Gem and Nab in PDAC patients. The MTD was established to 200 mg SCO-101 when combined with 80% of the recommended dose of Gem and Nab, and the treatment was generally tolerable with manageable toxicity. In the highest SCO-101 cohort of 250 mg (cohort 5), the observed DLT was thrombocytopenia, which is a well-known side effect to Gem and Nab [5, 25], but not to SCO-101 [18]. Additional toxicities included hyperbilirubinemia and mild headache, which is in accordance with previously reported side effects of SCO-101 as a single drug [18]. The hyperbilirubinemia was transient and normalized before next planned dosing of SCO-101, and hyperbilirubinemia was not accompanied by a rise in liver enzymes or clinical symptoms besides jaundice in three patients. Allowing one cycle without the experimental drug in five patients might, however, have influenced the toxicity assessment of the combination.

The pharmacokinetics for SCO-101 assessed in the present study was in full agreement with previously published data in healthy volunteers [18]. We found a proportional relationship between doses and C_max_ and AUC_0-8 h_. These data verified that SCO-101 was accumulated in the plasma of patients before chemotherapy.

Efficacy was a secondary endpoint. The response, PFS, and OS results in this heterogeneous primarily second-line population are in line with published results of prospective phase II or cohort studies of Gem and Nab in second-line settings, with ORR ranging from 3.1 to 17.5%, mPFS ranging from 2.7 to 5.8 months, and mOS ranging from 6.6 to 9.9 months [26–28].

SCO-101 has previously been studied together with chemotherapy in patients with colorectal cancer, where the compound counteracts resistance to irinotecan by inhibiting the drug efflux pump ABCG2 and the metabolic liver enzyme UGT1A1 [8]. The recently completed phase II study (CORIST) of SCO-101 together with 5-flourouracil and irinotecan (FOLFIRI) in patients with prior progression on FOLFIRI (NCT04247256) showed tumor response in 4 of 25 patients (16%), and a mOS of 10.4 months [29]. In that study, blood bilirubin was suggested as a potential biomarker for SCO-101. Used together with irinotecan, SCO-101 led to increased neutropenia due to SCO-101-mediated UGT1A1-inhibition and potentiation of irinotecan [29]. In the present study, we found no indications of a similar neutropenic effect, which is consistent with Gem and paclitaxel not being substrates for UGT1A1 [10].

Potential mechanisms of chemoresistance and preclinical evidence of resistance-modifying drugs in PDAC have been reviewed in numerous prior reports [30–33]. Unfortunately, the outcomes of such compounds in clinical studies have been disappointing. For example, celecoxib, a selective COX-2 inhibitor that may potentiate the growth-inhibitory effects of chemotherapeutic agents, was combined with Gem and cisplatin in a clinical phase II study, but no apparent effect was seen [34]. In another phase II trial, genistein, an isoflavone that may inactivate Akt and NF-κB and enhance the anti-tumor activity of erlotinib and Gem, showed no clear effect on survival of PDAC patients when added to the chemotherapy [35]. Recently, irbesartan, an angiotensin type 1 receptor antagonist used for treating hypertension, was shown to overcome Gem resistance in PDAC models by suppressing stemness and iron metabolism via inhibition of the Hippo/YAP1/c-Jun axis. Clinical activity of the drug was suggested in two small retrospective cohorts [36].

Due to its blocking of the ABCG2 efflux pump, SCO-101 has potential impact on resistance mechanisms of several antineoplastic drugs [37]. Overexpression of ABCG2 is regarded as one of the main causes of multi-drug resistance [37], and the ABCG2 gene expression level is increased in PDAC tissue compared to adjacent non-cancer tissue [38]. Exposure to Gem can increase ABCG2 levels [12] and increased levels of ABCG2 in Gem-resistant PDAC cells have been shown [39] as well as high ABCG2 gene expression in tumors was associated with reduced response rate and shorter PFS in PDAC patients treated with Gem and Nab [40]. However, neither paclitaxel nor Gem is a classical substrate for ABCG2 [9], and our preclinical study showed that blocking ABCG2-activity in PANC-1-Pac cells did not significantly increase the sensitivity to paclitaxel. In a second ABCG2-positive cancer model, we also showed that there was no combination effect of ABCG2-inhibition and paclitaxel or Gem.

Another important member of the drug efflux family of ABC transporters is ABCB1, which is known to efflux paclitaxel among many other drugs [41]. We showed that inhibition of ABCB1 in the PANC-1-Pac cells increased the sensitivity to paclitaxel. However, with a demonstrated high IC50 value of 16.1 µM, SCO-101 is not a strong inhibitor of ABCB1. SCO-101 also inhibits UGT1A1 but neither Gem nor paclitaxel is known as a substrate [10], and UGT1A1 is not present in pancreatic cells [42].

We could confirm resent results that SCO-101 inhibits SRPK1 [16]. We also found that SCO-101 and a highly selective SRPK1-inhibitor (SRPIN340) both caused synergistic effect with paclitaxel in paclitaxel-resistant PDAC cells. SRPK1 has been implicated in all the cancer hallmarks, SRPK1 expression correlates with advanced disease and poor survival in several cancer indications [15, 43], its expression is elevated in malignant and dysplastic pancreatic tissue compared to normal pancreatic tissue, and, in PDAC cells, SRPK1 silencing inhibits proliferation, induces apoptosis, and enhances the sensitivity to Gem and cisplatin [14]. Hence, the mechanism of SCO-101 in reversing resistance to taxanes or Gem is not likely to involve UGT1A1 or ABCB1 inhibition but may involve inhibition of SRPK1 and/or ABCG2. Other resistance mechanisms to Gem and taxanes include nucleoside transporters, microenvironmental factors, and microtubule proteins [31, 44] and therefore it would be of interest to further explore if SCO-101 targets any of these.

Study limitations include the lack of preclinical in vivo data. Hence, the study was embarked due to results of a single pre-clinical data set from a single paclitaxel-resistant cell line. No data on target engagement was included; however, preclinical data shows that SCO-101 inhibits UGT1A1 [45], and the rise in plasma bilirubin observed with treatment with SCO-101 also strongly suggests that the drug blocks cellular transport mechanisms associated with bile excretion.

In conclusion, we demonstrate preclinical evidence that SCO-101 acts synergistically and increases cell killing together with paclitaxel in human paclitaxel-resistant PDAC cells, possibly by inhibition of SRPK1 and/or ABCG2. In patients with non-resectable or metastatic PDAC, the combination of oral SCO-101 with Gem and Nab was well tolerated. Transiently increased blood bilirubin and mild headache were the main toxicities attributable to SCO-101. The MTD was determined to be 200 mg SCO-101 daily for 6 days on a bi-weekly schedule together with Gem and Nab. In this small study, we found no clear indications of added efficacy of the regimen.

## Supplementary Information

Below is the link to the electronic supplementary material.

Supplementary file1 (DOCX 2776 KB)

## Data Availability

The datasets generated during and/or analyzed during the preclinical study are available from the corresponding author on reasonable request.

## Abbreviations

ABC: ATP-binding cassette
AE: Adverse event
AUC: Area under the plasma drug concentration–time curve
Ca: Cancer antigen
CBR: Clinical benefit rate
CI: Confidence interval
C_max_: Maximum drug plasma concentration
CR: Complete response
CTCAE: Common Terminology Criteria for Adverse Events
DLT: Dose-limiting toxicity
ECOG: Eastern Cooperative Oncology Group
Gem: Gemcitabine
G-CSF: Granulocyte-colony stimulating factor
HR: Hazard ratio
HV: Healthy volunteers
IC50: Half-maximal inhibitory concentration
mOS: Median overall survival
mPFS: Median progression-free survival
MTD: Maximum tolerated dose
Nab: *Nab*-Paclitaxel
ORR: Objective response rate
PD: Progressive disease
PDAC: Pancreatic ductal adenocarcinoma
PK: Pharmaco-kinetics
PR: Partial response
PS: Performance status
SAE: Serious adverse event
SD: Stable disease
SRPK1: Serine-arginine protein kinase 1
SUSAR: Suspected unexpected serious adverse reaction
T_½_: Half-life
TTP: Thrombotic thrombocytopenic purpura
UGT: UDP glucuronosyltransferase
ULN: Upper limit of normal
ZIP: Zero interaction potency
