# Supplementary material for: PANTAX: a phase Ib clinical trial of the efflux pump inhibitor SCO-101 in combination with gemcitabine and nab-paclitaxel in non-resectable or metastatic pancreatic cancer
Source: Invest New Drugs. 2025 Apr 24;43(2):337–47. doi: 10.1007/s10637-025-01526-7 (PMC12048447; doi:10.1007/s10637-025-01526-7)
Supplement: Supplementary file 1 — Supplementary file1 (DOCX 2776 KB) [file 10637_2025_1526_MOESM1_ESM.docx]

## Supplementary materials

**Preclinical study materials and methods**

A paclitaxel-sensitive human PDAC cell line PANC-1 was obtained (ATCC, CRL-1469) and the derived paclitaxel-resistant PANC-1-Pac cell line was established by gradually increasing concentrations of paclitaxel over a period of 2-4 months. Standard MTT and crystal violet assays were applied to evaluate the effect of treatment with either combinations of SCO-101/paclitaxel or SRPK1 inhibitor/paclitaxel.

The selection began at 1 nM, increased to 2 nM and 4 nM, and ended at 8 nM. For each drug concentration the cells were kept for three passages. To evaluate the effect of the treatment either a Crystal Violet assay or MTT (3-(4,5-dimethylthiazol-2-yl)-2,5-diphenyltetrazolium bromide) assay were applied.

The MTT was conducted according to standard conditions. In short, cells were seeded at a density of 7,000 cells/well into a 96-well. One day after seeding, the cells were experimentally treated. Control conditions consisted of either growth medium (‘untreated’) or DMSO diluted in growth medium. All treatments were performed in triplicates. The cells were then kept in the CO_2_ incubator at 37˚C for 72 hours and plates were incubated with 100μl 1:10 MTT (0.5 mg/ml dissolved in phosphate buffered saline) for 3 hours and hereafter stopped using 100μl/well stop buffer (50g Sodium Dodecyl Sulphate, 245 mL MiliQ H_2_O, 5mL 1.0 N HCl was added to dissolve the formazan crystals overnight. Optical densities were measured at 570 and 670 nm.

For the crystal violet assay, cells were seeded into 6-well plates at a density of 40,000 cells/well or into 48-wells with 4000 cells/well. One day after seeding, the cells were experimentally treated. Control conditions consisted of either growth medium (‘untreated’) or DMSO diluted in growth medium. All treatments were performed in triplicates. The cells were then kept in the CO_2_ incubator at 37˚C until staining with crystal violet dye for 10mins and let to dry overnight. The following day crystal violet dye was dissolved using 20% acetic acid and absorbance was measured at 570 nm.

Experiments with 4 different concentrations of either SCO-101 or paclitaxel and their combinations were applied to test for synergy among these two compounds in both the PANC-1 parental and -Paclitaxel resistant cells. SCO-101 was applied in 10, 20, 40 and 50 µM whereas paclitaxel was tested in 5, 10, 15 and 20 nM for a total of 144 h, where drug was only present in the first 72h (sFig 2). The SRPK1 inhibitor SPHINX31 was tested at 0.1; 0.5; 1 and 5 µM. The SRPK1 inhibitor SRPIN was tested at 1; 5; 10 and 50 µM. Both inhibitors were tested in combination with 5, 10, 15 and 20 nM Paclitaxel.

Experiments with the HT29-SN38 resistant cell lines was carried out according to the experimental protocols described in [REF til Jensen et al. PMID 25759163].

Metabolism of paclitaxel and gemcitabine were investigated using incubations with hepatic human recombinant UGT1A1 (Baculovirus expressed recombinant human UGT enzymes from Corning): Controll supersomes were used as negative controls. The incubations were conducted using 2 μM or 20 μM initial concentrations of paclitaxel or gemcibatine for a 60 min incubation period and 0.3 mg/ml protein concentration for recombinant UGT1A1. Samples were analyzed using U-HPLC/QE-Orbitrap-MS.

Formation of direct glucuronide conjugate of paclitaxel and gemcitabine was monitored in the 60 min incubation with recombinant human UGT1A1. Assays were conducted as per the manufacture’s recommendation.

Analysis of SCO-101 ABCB1 (MDR1) efflux transporter inhibition was evaluated by measuring the vesicular uptake of a probe substrate into inside-out membrane vesicles expressing ABCB1 (MDR1) human ABC transporter in the absence and presence of SCO-101 (1.2 –300 μM) with and without ATP. Transporter inhibition was observed as reduced level of vesicular uptake of the probe substrate in the presence of ATP. ABCB1 (MDR1) assay was conducted with reagents from PredivezTM VT Reagent Kit for MDR1/P-gp (SOLVO Biotechnology). Assays were conducted as per the manufacturer’s recommendation.

**Supplementary table 1. Inclusion and exclusion criteria of patients in the Pantax phase Ib study.**

| **Inclusion criteria** | **Exclusion criteria** |
| --- | --- |
| 1. Ability to understand and willingness to provide written informed consent before any trial-related activities. 2. Age 18 years or older. 3. Histologically or cytologically verified pancreatic adenocarcinoma. 4. Inoperable localized, locally advanced or metastatic pancreatic cancer, not amenable for curatively intended treatment, in patients who are to be treated with Gem and Nab. 5. Measurable or non-measurable disease determined by CT scan or MRI, according to RECIST 1.1. 6. ECOG PS ≤ 2 and expected to tolerate the standard recommended (100%) Gem and Nab dose. 7. Recovered to grade 1 or less from prior surgery or acute toxicities of prior radiotherapy or treatment with cytotoxic or biologic agents. 8. ≥ 2 weeks elapsed since any prior surgery or radiotherapy. 9. Adequate conditions as evidenced by the following clinical laboratory values*: ANC ≥ 1.5 x 10^9^ /L, Hemoglobin ≥ 6.0 mmol/L, Platelets ≥ 100 x 10^9^ /L, ALT ≤ 2.5 x ULN and AST ≤ 2.5 x ULN*, Total blood bilirubin ≤ 1.0 ULN, Alkaline phosphatase ≤ 2.5 x ULN, Creatinine ≤ 1.5 ULN, eGFR within normal limits, Adequate blood clotting function as defined by INR < 1.5. 10. Life expectancy longer than 3 months. 11. Sexually active males and females of child-producing potential must use highly effective contraception (intrauterine devices, hormonal contraceptives (contraceptive pills, implants, transdermal patches, hormonal vaginal devices or injections with prolonged release) for the study duration and at least 6 months after the last dose of study drug. 12. Signed informed consent. | 1. Concurrent chemotherapy, radiotherapy, or other investigational drug during study period. One cycle of chemotherapy with Gem and Nab is allowed before signing the informed consent form and entering the study^+^. 2. Previous surgeries with resection of the complete stomach or greater part of small intestines (excluding the duodenum), 3. Difficulty in swallowing tablets. 4. CNS metastases requiring steroids. 5. Treatment with antibiotics for infections or with clinical symptoms of active infection. Patients showing symptoms of CoViD19 must be tested for active CoViD19 infection^+^. 6. Known HIV positivity. 7. Known active hepatitis B or C. 8. Clinically significant cardiovascular disease: Stroke, Transient ischemic attack or myocardial infarction within ≤ 6 months prior to day 1. Unstable angina or NYHA Grade II or greater congestive heart failure. Serious cardiac arrhythmia requiring medication. 9. Mental status, symptomatic epilepsy or other CNS disease where the investigator assesses the patient not fit for the clinical study. 10. Other medications or conditions that in the Investigator’s opinion would contraindicate study participation of safety reasons or interfere with the interpretation of study results. Other severe medical conditions, including serious heart disease, unstable diabetes, uncontrolled hypercalcemia or previous organ transplants. Participation in another clinical trial with experimental medication within 30 days prior to registration. 11. Known hypersensitivity to Gem and/or Nab. 12. Pregnant women or women who are breastfeeding. 13. Prior or present neuropathy > grade 1. 14. Curatively intended treatment. |

*AST not mandatory. In case of known liver metastases with ALT and AST ≤ 5 x ULN and/or alkaline phosphatase ≤ 5 x ULN: Patients who do not conform to the transaminase and/or alkaline phosphatase inclusion criteria, but who by the principal investigator are considered in good PS and otherwise eligible for inclusion, and where the transaminase and/or alkaline phosphatase levels are considered elevated due to other reasons than deteriorated lever capacity, may be considered for inclusion based on conferred agreement between PI and sponsor.

ANC, absolute neutrophils count; AST, aspartate aminotransferase; ALT, alanine aminotransferase; eGFR, estimated glomerular filtration rate; Gem, gemcitabine; INR, International Normalized Ratio; Nab, *nab*-paclitaxel; NYHA, New York Heart Association; PS, performance status; ULN, upper limit of normal range.

^+^amended.

**Supplementary table 2S. Clinical characteristics of 22 included patients.**

|  | Number of patients |
| --- | --- |
| Gender | |
| Male | 11 |
| Female | 11 |
| Ethnicity | |
| Caucasian | 22 |
| Smoking status |  |
| Never smoker | 12 |
| Current smoker | 3 |
| Former smoker | 7 |
| Disease stage at inclusion | |
| Locally advanced or locally recurrent | 3 |
| Metastatic | 19 |
| Primary tumor site | |
| Head or uncinate process | 12 |
| Neck, corpus or tail | 3 |
| Data missing | 7 |
| ECOG PS | |
| 0 | 16 |
| 1 | 5 |
| 2 | 1 |
| Body Mass Index | |
| Elevated | 7 |
| Normal (18.5-24.9) | 13 |
| Decreased | 2 |
| Serum Ca 19-9 | |
| Normal | 4 |
| Elevated (median; range) 848; 43-13,700 U/ml | 15 |
| Data missing | 3 |
| Prior curative pancreatic surgery | |
| No | 14 |
| Yes | 8 |
| Prior treatment regimens completed (multiple possible) | |
| FOLFIRINOX | 16 |
| Gemcitabine and 5-FU/FU-analogue | 3 |
| Irinotecan and 5-FU | 3 |
| Gemcitabine and *nab*-paclitaxel | 1 |
| Gemcitabine | 2 |
| Onivyde pegylated liposomal | 1 |
| 5FU/FU-analoge | 1 |
| None | 5 |
| Prior pancreatic radiotherapy or chemoradiotherapy | |
| Yes | 7 |
| No | 15 |

Ca, Cancer antigen; ECOG PS, Eastern Cooperative Oncology Group performance status; FU, fluorouracil.


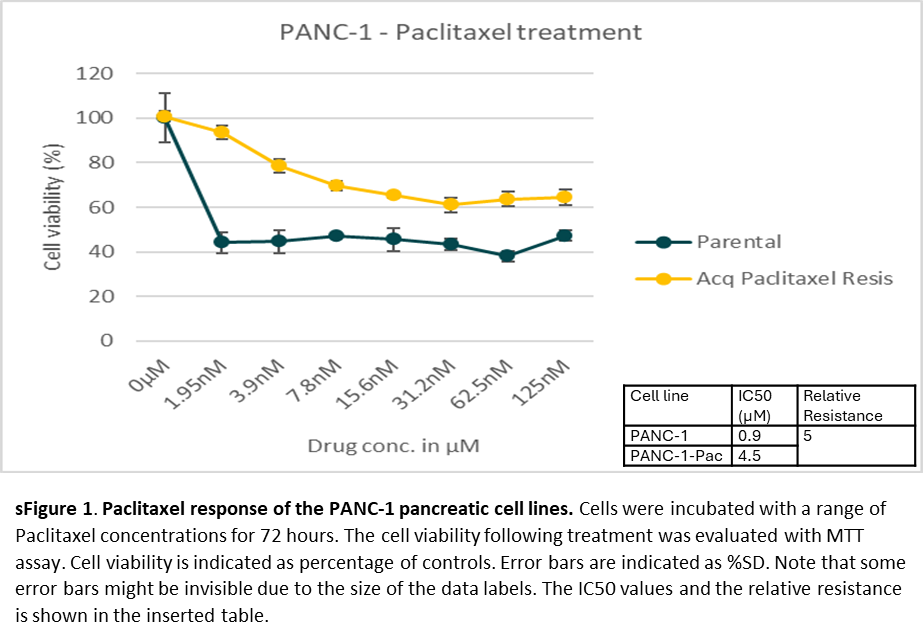


**Supplementary figure 1. Paclitaxel** **response of the PANC-1 pancreatic cell lines.**

Cells were incubated with a range of paclitaxel concentrations for 72 hours. The cell viability following treatment was evaluated with MTT assay. Cell viability is indicated as percentage of controls. Error bars indicate percentage standard deviation. Note that some error bars might be invisible due to the size of the data labels. The IC50 values and the relative resistance is shown in the inserted table.

**PANC-1-Pac PANC-1**


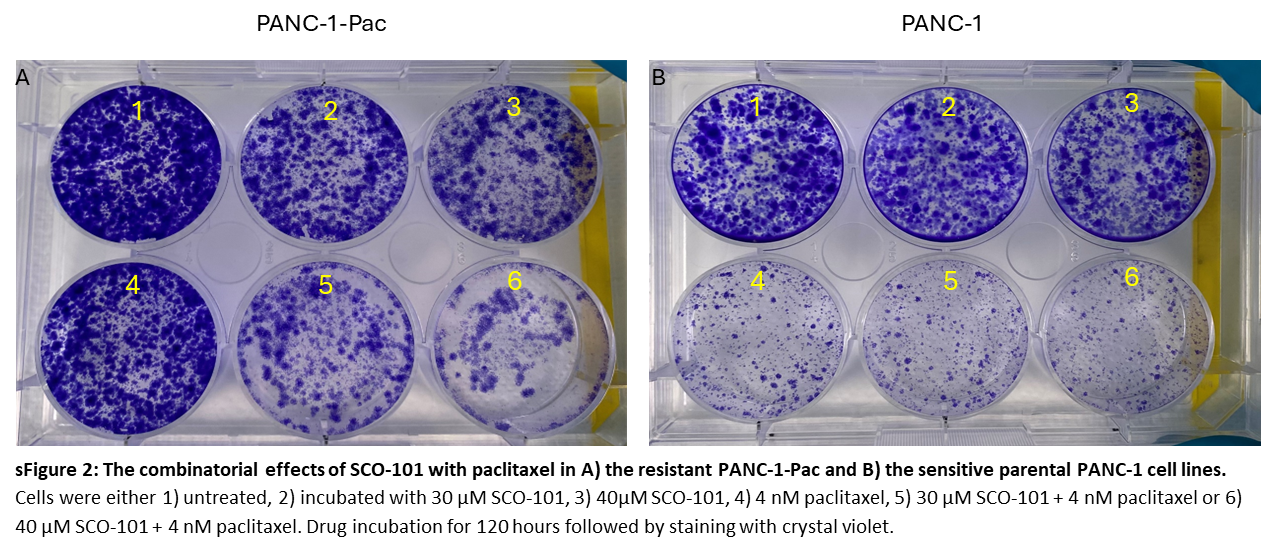


**Supplementary figure 2. The combinatorial effects of SCO-101 with paclitaxel.**

A) the resistant PANC-1-Pac and B) the sensitive parental PANC-1 cell lines. Cells were either 1) untreated, 2) incubated with 30µM SCO-101, 3) 40µM SCO-101, 4) 4nM paclitaxel, 5) 30µM SCO-101 + 4nM paclitaxel or 6) 40µM SCO-101 + 4nM paclitaxel. Drug incubation for 120 hours was followed by staining with crystal violet.


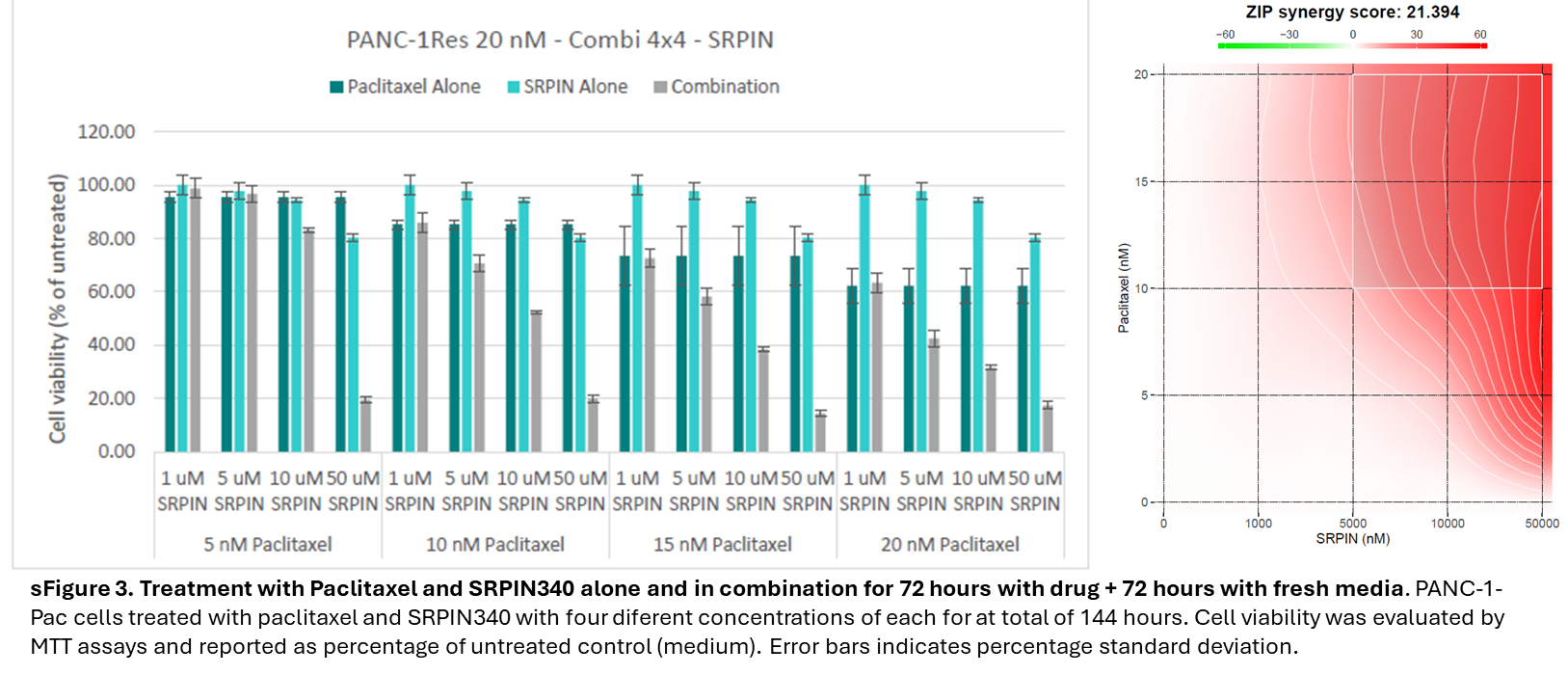


**Supplementary figure 3. Treatment with paclitaxel and SRPIN340 alone and in combination for 72 hours with drug + 72 hours with fresh media**.

PANC-1-Pac cells treated with paclitaxel and SRPIN340 with four different concentrations of each for a total of 144 hours. Cell viability was evaluated by MTT assays and reported as percentage of untreated control (medium). Error bars indicate percentage standard deviation.

**Disappearance of paclitaxel at 2 and 20µM**

**Disappearance of gemcitabine at 2 and 20µM**

**Supplementary figure 4. Metabolism of paclitaxel or gemcitabine using incubations with hepatic human recombinant UGT1A1.**

A) Mean disappearance of paclitaxel (2 and 20µM initial concentrations) as remaining LC/MS peak area % related to 0 min (N=2). B) Mean disappearance of gemcitabine (2 and 20µM initial concentrations) as remaining LC/MS peak area % related to 0 min (N=2).

Panc-1-Pac cells Panc-1-Pac cells

**Supplementary figure 5. Effect of 72 hours paclitaxel treatment in combination with Ko143 and zosuquidar plus 72 hours with medium alone in the PANC-1-Pac cell line.**

A) Cell viability of cells treated with 1µM Ko143 alone, paclitaxel alone (5, 10, 15 or 20nM) and in combinations. B) Cell viability of cells treated with 1µM zosuquidar alone, paclitaxel alone (5, 10, 15 or 20nM) and in combinations. Data is presented as percentage normalized to untreated cells. Error bars indicate standard deviations.

**HT29_SN38-Res_ Cells HT29_SN38-Res_ Cells**

**Supplementary figure 6. Effect of 72 hours paclitaxel or gemcitabine treatment in combination with Ko143 in ABCG2-positive SN38-resistant colorectal cancer cells (HT29-SN38-res).**

A) HT29-SN38-res treated with Ko143 alone, paclitaxel alone and in combinations. B) HT29-SN38-res treated with Ko143 alone, gemcitabine alone and in combinations. Data is presented as percentage normalized to untreated cells. Error bars indicate standard error of the mean.


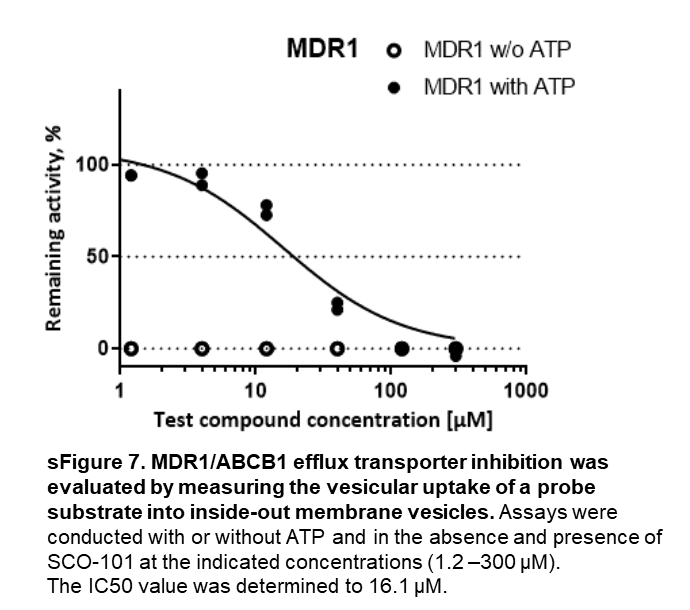


**Supplementary figure 7. SCO-101-inhibition of MDR1/ABCB1 efflux transporter**.

The inhibition was evaluated by measuring the vesicular uptake of a probe substrate into inside-out membrane vesicles. Assays were conducted with or without ATP and in the absence and presence of SCO-101 at the indicated concentrations (1.2 –300μM). The IC50 value for SCO-101-inhibition of MDR1/ABCB1 was determined to 16.1µM.
